# Increasing Realism and Variety of Virtual Patient Dialogues for Prenatal Counseling Education Through a Novel Application of ChatGPT: Exploratory Observational Study

**DOI:** 10.2196/50705

**Published:** 2024-02-01

**Authors:** Megan Gray, Austin Baird, Taylor Sawyer, Jasmine James, Thea DeBroux, Michelle Bartlett, Jeanne Krick, Rachel Umoren

**Affiliations:** 1 Division of Neonatology University of Washington Seattle, WA United States; 2 Division of Healthcare Simulation Sciences Department of Surgery University of Washington Seattle, WA United States; 3 Department of Family Medicine Providence St Peter Olympia, WA United States; 4 Department of Pediatrics Children's Hospital of Philadelphia Philadelphia, PA United States; 5 Department of Pediatrics San Antonio Uniformed Services Health Education Consortium San Antonio, TX United States

**Keywords:** prenatal counseling, virtual health, virtual patient, simulation, neonatology, ChatGPT, AI, artificial intelligence

## Abstract

**Background:**

Using virtual patients, facilitated by natural language processing, provides a valuable educational experience for learners. Generating a large, varied sample of realistic and appropriate responses for virtual patients is challenging. Artificial intelligence (AI) programs can be a viable source for these responses, but their utility for this purpose has not been explored.

**Objective:**

In this study, we explored the effectiveness of generative AI (ChatGPT) in developing realistic virtual standardized patient dialogues to teach prenatal counseling skills.

**Methods:**

ChatGPT was prompted to generate a list of common areas of concern and questions that families expecting preterm delivery at 24 weeks gestation might ask during prenatal counseling. ChatGPT was then prompted to generate 2 role-plays with dialogues between a parent expecting a potential preterm delivery at 24 weeks and their counseling physician using each of the example questions. The prompt was repeated for 2 unique role-plays: one parent was characterized as anxious and the other as having low trust in the medical system. Role-play scripts were exported verbatim and independently reviewed by 2 neonatologists with experience in prenatal counseling, using a scale of 1-5 on realism, appropriateness, and utility for virtual standardized patient responses.

**Results:**

ChatGPT generated 7 areas of concern, with 35 example questions used to generate role-plays. The 35 role-play transcripts generated 176 unique parent responses (median 5, IQR 4-6, per role-play) with 268 unique sentences. Expert review identified 117 (65%) of the 176 responses as indicating an emotion, either directly or indirectly. Approximately half (98/176, 56%) of the responses had 2 or more sentences, and half (88/176, 50%) included at least 1 question. More than half (104/176, 58%) of the responses from role-played parent characters described a feeling, such as being scared, worried, or concerned. The role-plays of parents with low trust in the medical system generated many unique sentences (n=50). Most of the sentences in the responses were found to be reasonably realistic (214/268, 80%), appropriate for variable prenatal counseling conversation paths (233/268, 87%), and usable without more than a minimal modification in a virtual patient program (169/268, 63%).

**Conclusions:**

Generative AI programs, such as ChatGPT, may provide a viable source of training materials to expand virtual patient programs, with careful attention to the concerns and questions of patients and families. Given the potential for unrealistic or inappropriate statements and questions, an expert should review AI chat outputs before deploying them in an educational program.

## Introduction

Virtual standardized patients (VSPs) represent an emerging technology with the potential to revolutionize health care education and training. They provide health care professionals with a safe and controlled environment in which to learn and practice complex skills. VSPs are frequently used in educational models for the health professions to teach history-taking, surgical skills, decision-making, and medication management [1-4]. VSPs have also been used in the health professions to practice critical communication skills [5-7]. VSPs that use natural language processing may provide a valuable educational experience for learners [8].

One example of a VSP is VANESSA (Virtual Antenatal Encounter and Standardized Simulation Assessment) [9]. The VANESSA simulator is a screen-based simulation of a woman in her 23rd week of gestation who can display multiple emotions through the animation of facial expressions and body language. The VANESSA simulator was developed by the Neonatal Education and Simulation-Based Training Laboratory at the University of Washington to teach prenatal counseling skills to residents and fellows [9]. In its initial iteration, VANESSA was given a list of manually generated responses that neonatologists who routinely do perinatal counseling deemed relevant and realistic to the conversation. Manually generating a large, varied sample of realistic and appropriate parent responses for VANESSA has been challenging. Unrealistic responses and questions reduce the fidelity of virtual simulations. Newly developed artificial intelligence (AI) systems can provide dialogue for a wide variety of interactions and may be a valuable resource in expanding virtual patient dialogues for specific clinical scenarios, such as prenatal counseling.

Chat-based language models and AI are entering the public domain with impressive performance, a large application pool, and exciting interactivity. Notably, ChatGPT has prompted a billion-dollar investment from Microsoft, triggered explicit discussions by Bill Gates and Elon Musk, and captivated the population of users able to interact with it via the open research chat interface. AI trained with large language models to interpret written or auditory input and generate coherent, domain-centered responses is being proposed in a variety of real-world applications, including the health care setting. ChatGPT has the added benefit of being able to emulate different characters, allowing for a broader array of parent voices than could be generated by individual health care educators.

In this report, we explore the use of ChatGPT to enhance the realism of the VANESSA VSP. We hypothesized that the integration of the ChatGPT AI chatbot would generate realistic, relevant, and usable patient responses for a VSP simulator used in prenatal counseling education.

## Methods

The study used an exploratory observational design, with ChatGPT acting as an expectant parent within the VANESSA software, conducted in February 2023 on ChatGPT 3.5.

The VANESSA VSP represents a pregnant woman in her 23rd week of gestation and showcases emotions through animated facial expressions and body language. Created with input from neonatologists, its dialogue and emotive feedback were found realistic in pilot tests, enabling participants to confidently identify its emotional states.

ChatGPT is a large language model developed by OpenAI. Its exceptional performance stems from generative pretraining, leveraging extensive unlabeled data sets [10]. This foundational training helps it grasp English nuances. Following this pretraining is “one-shot” learning, a rapid task-specific learning [11]. The architecture includes a transformer encoder-decoder neural network, originally developed for translation services and now popular in language models [12]. ChatGPT decodes user prompts to create relevant responses using autoregressive language modeling [13]. It is apt for generating realistic dialogue for health care simulations.

**Figure 1:**
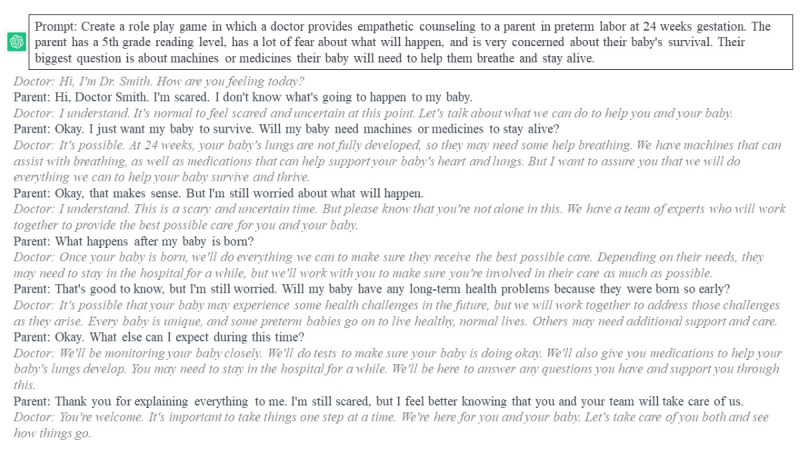
Example role-play conversations.

The study had three phases:

1. ChatGPT generated a list of common concerns from families expecting preterm delivery at 24 weeks. The stability of these concerns was verified in an iterative process over time.
2. Using a standardized prompt, ChatGPT crafted potential parent questions related to each concern.
3. Role-plays were constructed for a mother expecting preterm delivery. The AI was given varied emotional settings for the scenario of preterm labor at 24 weeks, including anxiety or distrust in the medical system. In each scenario, a designated area of concern and a primary question were specified, derived from the potential parent responses generated in phases 1 and 2. Conversations were created with cues for the VSP and then reviewed (an example is shown in Figure 1).

Throughout the process, ChatGPT was instructed to adhere to a fifth-grade reading level for the AI parent role. Considering the US Department of Education’s findings on widespread low literacy, the importance of health literacy, and the impacts of pain, stress, and other factors on comprehension, this was deemed crucial [14-17]. The Joint Commission and several medical organizations suggest that patient materials should be at a fifth-grade level or lower [18]. Although these dialogues were verbal, the principle of understandability remained in place.

Role-play conversations were scrutinized for parental responses. Each was checked for question or statement content, emotional cues, and sentence count. Initially, generated physician names and certain response starters (eg, “yes” or “no”) were noted but removed for evaluation. Sentences were then appraised by a neonatologist for realism, relevance, and usability for virtual prenatal counseling simulations. Each metric used a 5-point Likert scale, ranging from 1 (the lowest) to 5 (the highest). For usability in the VANESSA VSP, responses were scored as follows: 1 if they were unusable, 2 if they were unusable without major modifications, 3 if they were usable with moderate modifications, 4 if only minor modifications were needed, and 5 if they were usable without any modifications. The first 10% of responses were independently reviewed by 2 experienced neonatologists (RU and MG) and then compared for reliability. A calculated weighted kappa on the sample was 0.84, which is considered a strong level of agreement [19]. Responses with differences in rating were discussed by the team members to improve reliability, and the remainder of the data set was scored by one of the experienced neonatologists. Duplicate responses were scored only once. Analysis was done using Stata (version 17.0; StataCorp).

## Results

ChatGPT-3.5 generated a list of 7 common areas of concern, 28 questions likely to be asked by parents anxious about the preterm delivery of their infant, and 7 additional questions likely to be asked by parents with low trust in the medical system (Table 1). These areas of concern and questions were used to create 35 unique role-plays, which contained 176 unique parent responses (Table 2). The role-plays had a median of 5 (IQR 4-6) parent responses to the counseling physician. The responses were roughly evenly split between questions and statements. About half of the responses had 2 or more sentences in the response. Many responses mentioned a specific emotion or feeling. The role-play of the parent with low trust in the medical system generated 50 unique sentences across the 7 areas of concern. There was variation in the number of unique sentences generated across the 7 major areas of concern (Table 3). Most responses were found to be realistic, appropriate for variable conversation paths, and usable in a VSP program (Table 4).

**Table 1:** Areas of concern and example questions generated by artificial intelligence.

Areas of concern	Example questions from parents
Health and development	Will our baby be healthy if they are born too soon? What will the doctors do to help our baby be healthy and strong? Can our baby get sick more easily if they are born too soon? Will the baby feel pain during birth or while in the hospital? I’m worried about the risks and complications, what if something goes wrong? (Mistrust)
Survival	Will the baby survive? What kind of help will our baby need to stay alive? How likely is it that our baby will survive? What kind of machines or medicines will our baby need to help them breathe and stay alive? I don’t know if I can trust the medical field, what are the chances of my baby surviving at 24 weeks? (Mistrust)
NICU^a^ stay	What is the NICU, and why does our baby need to go there? How long will our baby need to stay in the NICU? Can we visit our baby in the NICU, and how often? Will our baby be alone in the NICU, or will there be other babies and parents there too? What kind of things can we do to help our baby feel better in the NICU? Will anything happen in the NICU without my consent? (Mistrust)
Emotional impact	How do we get ready for having a baby born too soon? Can we hold and touch the baby in the hospital, and is this good for the baby? Who can help us if we are feeling sad or stressed about our baby being born too soon? I’m worried about my baby going to the NICU where she will be alone and scared (mistrust).
Long-term outcomes	What help can we get after we leave the hospital? Will our baby be able to do the same things as other babies who were born at the right time? Will our baby be okay in the future if they are born too soon? I don’t know what’s going to happen to my baby. I don’t really trust the doctors but what happens if my baby doesn’t develop properly? (Mistrust)
Feeding and nutrition	How will our baby get the right kind of food if they are born too soon? Can we feed our baby ourselves, or will they need special milk or formula? How often will our baby need to be fed, and how much? Will our baby be able to eat the same kinds of food as other babies when they get older? Can we breastfeed our preterm baby, or do we need to use formula? Will our baby be able to breastfeed right away, or will they need to be fed in a different way at first? Will I have any say in how my baby is fed? (Mistrust)
Quality of life	Will our baby be able to go to school and play sports like other kids? How can we help our baby if they have trouble learning or doing things in the future? What can we do to make sure our baby has the best chance for a good future? I’ve had bad experiences before and I’m scared about what’s going to happen to my baby in the future, what can I expect? (Mistrust)

^a^NICU: neonatal intensive care unit.

**Table 2:** Generated role-plays by artificial intelligence.

Characteristics		Values
**Role-plays (n=35), n (%)**		
	Worried about specific area of concern	28 (80)
	Low trust in the medical system	7 (20)
Responses per role-play, median (IQR)		5 (4-6)
**Parent responses (n=179), n (%)**		
	Unique responses	176 (98)
	Duplicate responses	3 (1)
**Types of responses (n=179), n (%)**		
	Statements	91 (51)
	Questions	88 (49)
**Sentences per response (n=179), n (%)**		
	1	81 (45)
	2	76 (42)
	3	18 (10)
	4	4 (2)
Duplicate sentences (n=305), n (%)		37 (12)
Total unique sentences (n=305), n (%)		268 (88)
**Feelings stated in responses (n=117), n (%)**		
	Specific emotion stated in phrase	56 (48)
	“Scared”	36 (31)
	“Worried	26 (22)
	“Anxious”	2 (2)
	“Concerned”	2 (2)
	“Afraid”	1 (1)
	“Nervous”	1 (1)
	“Overwhelmed”	1 (1)
	Emotion indirectly implied by phrase	51 (44)

**Table 3:** Sentences generated per role-play.

Area of concern	Number of unique sentences
Health and development	47
Survival	46
Feeding and nutrition	45
The NICU^a^ stay	40
Quality of life	36
Outcomes	28
Emotional impact	26

^a^NICU: neonatal intensive care unit.

**Table 4:** Ratings of relevance, realism, and usability of sentences generated by ChatGPT (N=254).

Characteristics	Rating, n (%)				
	1 (least)	2	3	4	5 (most)
Realism in parental responses and questions	5 (2)	8 (3)	38 (15)	20 (8)	183 (72)
Relevant to a prenatal counseling conversation	2 (1)	2 (1)	29 (11)	5 (2)	216 (85)
Usable for VSP^a^ educational program	5 (2)	1 (0)	87 (34)	34 (13)	127 (50)

^a^VSP: virtual standardized patient.

Modifications to responses were all aimed at ensuring the VSP could correctly deploy the phrase at the correct conversational juncture and that there were no elements of the phrase that might interrupt the flow. As ChatGPT 3.5 seeks to ensure the specific conversation has a flow, it can at times generate responses that are less usable for a VSP that needs to maintain flow across many different variations of the same conversation. Only 2% (5/254) of the AI-generated responses were not usable in the VSP. Examples of minimally usable responses included “How much should I feed my baby each time?” which is not relevant to how feeding is done in the neonatal intensive care unit and “I am,” as this response is too nonspecific to be of use in a VSP. Of the 34% (87/254) of responses that required moderate modifications, the changes primarily involved adjusting terminology to ensure the parent was using colloquial, jargon-free language. As an example, “I’ve been having a lot of contractions and I’m only 24 weeks pregnant” was modified to “I’ve been having a lot of cramping and am only 6 months pregnant.” Other modifications included adding some specificity to a response to ensure the VSP can use the sentence in the right context, such as modifying “That sounds reassuring, but what are the risks?” to “That sounds reassuring, but what are the risks of being born this early?” Of the 13% (34/254) of responses that required minimal adjustment, example changes included “I don’t trust the doctors” to “I don’t trust doctors,” and “Okay, thank you, but can you tell me more about what might happen to my baby in the future?” to “Can you tell me more about what might happen to my baby in the future?”

## Discussion

### Principal Findings

In this study, we examined the feasibility of using ChatGPT to enhance the realism of the VANESSA VSP. We found that the integration of ChatGPT generated many realistic, relevant, and useful responses. Based on these findings, ChatGPT-enabled VSPs may be beneficial in prenatal counseling education. There was more variation in realism and usability compared to relevance; therefore, an expert review was necessary to provide quality control before integrating the ChatGPT-generated conversations into an educational VSP program for prenatal counseling. Modifications made to responses to make them usable for the VANESSA VPS were largely focused on ensuring the virtual patient remains free of jargon and her responses maintain the flow of conversation.

Research conducted so far on AI chat engines has focused on using chat-based AI for the creation of discharge summaries, generating and interpreting electronic health records, assisting in medical education related to the medical licensing exam, and summarizing collections of journal articles to construct a brief abstract from the conclusions of the research [20-23]. The field is still relatively new, but rapidly increasing and expanding. This growth will only continue, as generating documentation and interacting with patients are key requirements of the health care setting. Health care simulation has many training applications, such as VSPs, that require expert authoring to educate clinicians and care providers on a certain skill or cognitive task. VSPs like VANESSA have been used in teaching the communication of medical ambiguity, evaluating medical students’ competence in performing critical clinical skills, and training nurses to recognize postpartum mood disorders [24-26]. Based on the results of our study, chat-based AI may be a valuable teaching tool in the future of health care simulation technology, leading to improved scenario creation, customization of patient interactions, and responses to care providers in a simulated setting. These improvements will result in authentic, unique interactive experiences, varying for each learner and training scenario.

We found that ChatGPT could generate many realistic parent responses, especially concerning issues related to survival at 24 weeks gestation and the neonatal intensive care unit stay (Figure S1 in Multimedia Appendix 1). Mistrust in the health care system is often encountered during stressful counseling conversations, and building the skill of responding to mistrust is crucial for physicians during their training [27]. Patients who express mistrust are less likely to engage with their health care team and care plan, and care is needed to proactively build trust during prenatal counseling [28-31]. Including opportunities for learners to respond to VSPs that express mistrust is one way to address this important counseling element, and ChatGPT provided a reliable mechanism to generate these phrases. Interestingly, the ChatGPT bot faced more challenges in generating realistic questions and responses about the emotional impact of preterm delivery and feeding. As these are frequently encountered topics of conversation in prenatal counseling, an expert review of these conversational elements remains a vital step before including them in an educational program.

ChatGPT produced responses that seemed relevant and appropriate to the context of prenatal counseling. Previous studies of prenatal counseling for extreme prematurity indicate that parents may ask questions about the likelihood of various outcomes, express a range of emotions, request engagement in shared decision-making, and express their parental roles and values [32,33]. Parents may express statements about their uncertainty, anxieties, and hope for the future [34]. This wide range of topics, emotions, and questions makes it challenging to ensure that chatbot-generated conversations remain appropriate to the educational goals of the VSP. Despite the risk of getting off-topic, we found that only 1% (2/254) of ChatGPT-produced responses were irrelevant to a counseling conversation, given a carefully worded role-play prompt. Although most responses were relevant, some topics, such as spirituality and shared decision-making, did not come up in the role-play conversations. Previous studies have demonstrated that providers perceive the importance of parents’ spirituality in their decision-making and infrequently discuss these spiritual beliefs with parents in antenatal consultations [35,36]. Further work exploring how families might express their spirituality or explore shared decisions would be needed to ensure these topics are included in a VSP [37-39].

Chatbot programs use machine learning to generate their responses; due to the nature of machine learning, there is an inherent risk that chatbots can generate factually incorrect information [40]. Given this risk, caution is warranted when using chatbots in health care settings, where misinformation can have a significant risk [41,42]. Developers are working to address these inaccuracies as they design the next generation of large language model chat programs; they have demonstrated improvements in ChatGPT-4’s success across a variety of standardized tests [43]. This study leverages the strengths of a natural language chatbot in its ability to generate conversation while avoiding the risks of obtaining inaccurate medical information. Most scripts created by ChatGPT were usable for our perinatal counseling virtual patient. We found about a third of chatbot-generated phrases needed modification before being able to be integrated into a VSP; therefore, it may not be feasible to directly use ChatGPT for educational role-play without having the quality control step of review by expert clinicians. However, as technology continues to grow, this will evolve, and each subsequent model should be evaluated for usability.

### Study Limitations

This exploratory study has several limitations. First, the pilot was done using ChatGPT 3.5, which is a single platform and is not representative of all chatbots. Later versions of ChatGPT have already been released and may have differences in realism, appropriateness, and usability. Newer AI chatbot programs are being trained on more parameters (175 billion for ChatGPT-3 vs an anticipated 100 trillion with ChatGPT-4), are supposed to have more ability to iterate on the same topic, and are being adjusted to improve the faculty accuracy of their responses [43]. Second, chatbot programs have limited information on which they build a conversation. For this study, we used a stable prompt around an impending 24-week gestation delivery to fit the standardized patient scenario, but conversations may be different with variations in the prompt. The AI was given a limited background to build a role-play, potentially limiting the diversity of ways in which patients could communicate their concerns. For this scenario, we requested a fifth-grade reading level for all patient roles to better mimic how patients may speak in stressful situations, but we did not explore higher or lower complexity of responses. Future work should explore how variations in the background, scenario, and reading level provided to the chatbot impact the output of the role-play. Another significant limitation was that response checking was performed by neonatologists, without input from families or trainees. Future work to refine the model will incorporate their views to ensure further applicability of the VSP and the validity of any assessments. Finally, although individual phrases exhibited good realism, the total duration of each patient-physician conversation (averaging 5 volleys) was generally shorter than that of a real prenatal counseling conversation.

### Conclusions

Generative AI programs, such as ChatGPT, may provide a viable source of training materials to expand VSP programs with careful attention to the concerns and questions of patients and families. Given the potential for unrealistic or inappropriate statements and questions, an expert should review AI chat outputs before deploying them in an educational program.

## Appendix

Multimedia Appendix 1 Realism in sentences generated by ChatGPT based on area of concern.

## Abbreviations

AI: artificial intelligence
VANESSA: Virtual Antenatal Encounter and Standardized Simulation Assessment
VSP: virtual standardized patient
