# Supplementary figures and images for: Increasing Realism and Variety of Virtual Patient Dialogues for Prenatal Counseling Education Through a Novel Application of ChatGPT: Exploratory Observational Study
Source: JMIR Med Educ. 2024 Feb 1;10:e50705. doi: 10.2196/50705 (PMC10870212; doi:10.2196/50705)

Figure S1. Realism in Sentences Generated by ChatGPT based on Area of Concern.


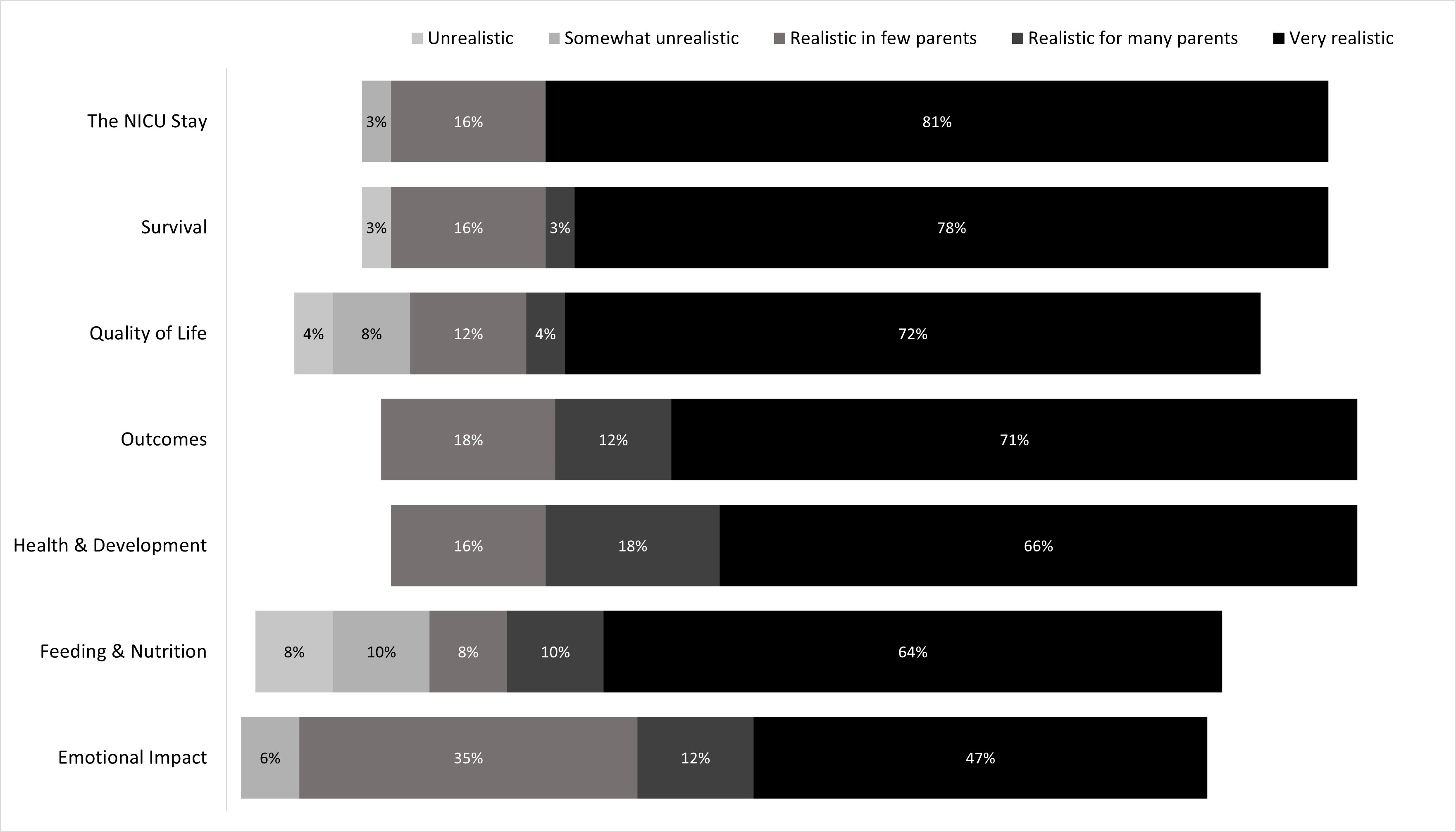

Supplement: Multimedia Appendix 1 [file mededu_v10i1e50705_app1.docx]
